# Supplementary material for: Identification of Antibacterial Components in the Methanol-Phase Extract from Edible Herbaceous Plant Rumex madaio Makino and Their Antibacterial Action Modes
Source: Molecules. 2022 Jan 20;27(3):660. doi: 10.3390/molecules27030660 (PMC8839378; doi:10.3390/molecules27030660)
Supplement: Supplementary file 1 [file molecules-27-00660-s001.zip › molecules-1501396-supplementary.pdf]

Table S1. Bacterial strains and media used in this study.

Bacterial strain	Culture medium	Source
Vibrio alginolyticus ATCC17749	TSB	ATCC, United States
Vibrio alginolyticus ATCC33787	TSB	ATCC, United States
Vibrio fluvialis ATCC33809	Marine 2216	ATCC, United States
Vibrio harvey ATCC BAA-1117	Marine 2216	ATCC, United States
Vibrio harveyi ATCC33842	Marine 2216	ATCC, United States
Vibrio metschnikovii ATCC700040	Marine 2216	ATCC, United States
Vibrio mimicus bio-56759	TSB	Biobw, China
Vibrio parahaemolyticus ATCC17802	TSB	ATCC, United States
Vibrio parahaemolyticus ATCC33847	TSB	ATCC, United States
Vibrio vulnificus ATCC27562	TSB	Biobw, China
Aeromonas hydrophila ATCC35654	TSB	ATCC, United States
Bacillus cereus A1-1	TSB	LS-SHOU, China
Enterobacter cloacae ATCC13047	TSB	Biobw, China
Enterobacter cloacae	LB	LS-SHOU, China
Escherichia coli ATCC8739	TSB	Biobw, China
Escherichia coli K12	TSB	IIM, China
Escherichia coli ATCC25922	LB	ATCC, United States
Enterobacter sakazakii CMCC45401	TSB	Biobw, China
Listeria monocytogenes ATCC19115	BHI	Biobw, China
Pseudomonas aeruginosa ATCC9027	TSB	Biobw, China
Pseudomonas aeruginosa ATCC27853	TSB	Biobw, China
Staphylococcus aureus ATCC25923	TSB	ATCC, United States
Staphylococcus aureus ATCC8095	TSB	ATCC, United States
Staphylococcus aureus ATCC29213	TSB	ATCC, United States
Staphylococcus aureus ATCC6538	TSB	ATCC, United States
Staphylococcus aureus ATCC6538P	TSB	ATCC, United States
Staphylococcus aureus	TSB	LS-SHOU, China
Shigella dysenteriae CMCC51252	TSB	GCCC, China
Salmonella choleraesuis ATCC13312	TSB	ATCC, United States
Shigella flexneri CMCC51572	TSB	GCCC, China
Shigella flexneri ATCC12022	TSB	ATCC, United States
Shigella flexneri CMCC51574	TSB	GCCC, China
Salmonella paratyphi-A CMCC50093	TSB	GCCC, China
Shigella sonnei ATCC25931	TSB	ATCC, United States
Shigella sonnet CMCC51592	TSB	GCCC, China
Salmonella typhimurium ATCC15611	TSB	ATCC, United States
Salmonella	LB	LS-SHOU, China
Vibrio parahaemolyticus B3-13	TSB	LS-SHOU, China
Vibrio parahaemolyticus B4-10	TSB	LS-SHOU, China
Vibrio parahaemolyticus B5-29	TSB	LS-SHOU, China
Vibrio parahaemolyticus B9-35	TSB	LS-SHOU, China
Vibrio cholerae GIM1.449	TSB	GCCC, China
Vibrio cholerae Q10-54	TSB	LS-SHOU, China
Vibrio cholerae b10-49	TSB	LS-SHOU, China

ATCC: American Type Culture Collection, United States; GCCC, Guangdong Culture Collection Center, Guangzhou, China; IIM, Institute of Industrial Microbiology, Shanghai, China; LS-SHOU, Laboratory stock, Shanghai Ocean University, Shanghai, China.

Table S2. Expression of representative DEGs by RT-qPCR assay.

Sample	Gene	Predicted protein	Fold change	
			RNA-Seq.	RT-PCR
V. alginolyticus ATCC17749	*N646_0236*	Hydroxylamine reductase	87.807	32.334
	N646_0310	Histidine ammonia-lyase	3.187	2.121
	N646_2909	Cation transport ATPase%2C E1-E2 family protein	0.123	0.142
	N646_4052	Putative acyl-CoA thiolase	5.154	1.288
	N646_4487	Arginine ABC transporter%2C periplasmic arginine-binding protein	4.958	7.863
V. parahaemolyticus ATCC17802	*VP_RS01760*	Dihydroxyacetone kinase ADP-binding subunit DhaL	0.040	0.111
	VP_RS01755	Dihydroxyacetone kinase subunit DhaK	0.067	0.158
	VP_RS05780	Hydroxylamine reductase	107.754	26.816
	VP_RS09370	Ammonia-forming nitrite reductase cytochrome c552 subunit	19.809	4.141
	VP_RS10480	Type I glyceraldehyde-3-phosphate dehydrogenase	0.168	0.011
	VP_RS10820	Chemotaxis protein CheA	0.386	0.498
	VP_RS16540	Flagellar basal body rod protein FlgB	0.064	0.667
	VP_RS22540	Flagellar biosynthesis protein FliQ	0.055	0.516
	VP_RS22500	Flagellar motor switch protein FliG	0.294	0.629
	VP_RS23260	6-phospho-beta-glucosidase	0.087	0.049
V. parahaemolyticus B4-10	*VP_RS05780*	Hydroxylamine reductase	107.754	10.740
	VP_RS06180	Histidine ammonia-lyase	6.284	1.860
	VP_RS06185	Urocanate hydratase	10.231	22.860
	VP_RS06190	Formimidoylglutamase	5.106	9.420
	VP_RS06195	Imidazolonepropionase	6.998	6.770
	VP_RS06485	ABC transporter ATP-binding protein	0.310	0.040
	VP_RS06520	ATP-binding cassette domain-containing protein	0.256	0.070
	VP_RS06525	ABC transporter permease subunit	0.265	0.130
	VP_RS20250	BC transporter permease	10.250	3.780
	VP_RS20670	ABC transporter ATP-binding protein	0.3698	0.018
	VP_RS20695	ABC transporter ATP-binding protein	0.455	0.022
B. cereus A1-1	*BCN_RS06525*	Indole-3-glycerol phosphate synthase TrpC	0.235	0.062
	BCN_RS08605	Flagellin	0.045	0.038
	BCN_RS08640	Flagellar type III secretion system pore protein FliP	0.108	0.557
	BCN_RS10010	Methyl-accepting chemotaxis protein	0.063	0.235
	BCN_RS10875	Hydroxylamine reductase	15.156	1.693
	BCN_RS16540	Respiratory nitrate reductase subunit gamma	150.78	233.631
